# Corruption in the News: Complicity in Canadian Newspaper Claimsmaking, 1980-2023

**DOI:** 10.1177/2631309X241242146

**Published:** 2024-04-01

**Authors:** Karl Guebert, Steven Bittle, Jon Frauley

**Affiliations:** 1 Department of Criminology, 6363 University of Ottawa, Ottawa, ON, Canada

**Keywords:** anti-corruption, corruption, discourse, news media, Organization for Economic Co-operation and Development

## Abstract

This paper explores the symbolic dimension of corruption in the print media, or the rhetoric deployed that reproduces a particular discursive order. Employing an abductive materialist analysis and drawing insight from the political-economy and critical anti-corruption(ism) literatures, we examined over 2300 news items on corporate corruption in five prominent Canadian newspapers. In addition to finding differing and contradictory notions of corruption, we identify three phases of (anti)corruption rhetoric that represent notable moments in the struggle over the meaning of corruption and how to curb it. The paper concludes that Canadian newspapers have reproduced the language and claims of powerful voices emanating from the international realm rather than scrutinizing these claims, resulting in an incoherent Canadian popular discourse on corruption.

## Introduction

According to Breit (2010, p. 619), while there are many studies on corruption “we still know little of how it is socially and discursively constructed.” This statement highlights an important distinction between the material, practical dimension of action and the symbolic (discursive or ideological) dimension pertaining to perception, meaning, and signification. These two domains imply one another as everything regarded as a problem contains both. The bulk of research on corruption and anti-corruption focuses on the practice of corruption and how to curb it. Contemplation of how corruption is or could be conceptualized is done toward better operationalizing the term to formulate anti-corruption measures. A fraction of the literature focuses on how corruption came to be designated and defined as problematic. That is, there is far less attention given to the epistemology and ontology of the category itself. The few scholars concerned specifically with news coverage of corruption (Berti, 2018; Bratu & Kažoka, 2018; Breit, 2010; Kramer, 2013; Mancini et al., 2017; Palau & Palomo, 2021) tend to concentrate on the production or construction of a dominant narrative or simply assume an oversight function for a free press, which serves to impugn corrupt activities. Our paper, by contrast, emphasizes the reproductive element of news media.

Our goal is to explore the symbolic dimension of corruption in the print media, or the rhetoric deployed that reproduces a particular discursive order. Although newspapers typically enjoy legitimacy as a civil society institution of critical oversight in capitalist democracies, many have argued (Herman & Chomsky, 1988; Hall et al., 2017 [1978]) that they represent important sites of struggle over meaning and play a key role in manufacturing consensus, particularly as they amplify select issues and obfuscate others. This positions newspaper outlets as key *reproducers* of discursive positioning being played out elsewhere and in this way are key sites of struggle as players attempt to instal and disseminate their own definitions of what is corrupt and how to respond. We take news rhetoric as symptomatic of this process of reproduction.

News media, in our case Canadian newspapers, represent one site of struggle over the designation and definition of corruption.^1^ But it is not the news media or journalists that are doing the struggling. Instead, as a *site* of struggle, news media are conduits for corruption-related publicity utilised by legal and political actors, business executives and managers, nation-states, and international agencies, including the Organization for Economic Co-operation and Development (OECD), World Bank (WB), and Transparency International (TI). Contestations over how to define corruption and what makes for effective anti-corruption measures filter through the media as the latter uncritically circulate differing and often incompatible meanings of the terms.

As part of a broader project on corporate corruption in Canada, we examined how Canadian newspapers treated ‘corruption’ – both the concept and activity – as well as reported on Canada’s anti-corruption strategies spanning from 1980 to 2023. As an early signatory to the OECD Anti-Bribery Convention, along with a close neighbour to the US where, as we shall see, much of the anti-corruption rhetoric originated, Canada is a good empirical exemplar for understanding the general sensibilities reflected in the news during a time in which concerns with the corruption ‘problem’ took root globally. Using the roots of the search terms, “corporate” and “corruption,” and limiting results to those that contained both terms within 25 words of each other, we identified over 2300 news items (stories, opinions, letters to editor) appearing in five prominent Canadian newspapers: the Globe and Mail, Toronto Star, National Post, Ottawa Citizen, and Hamilton Spectator.

There are two major insights from our analysis. First, we yielded several differing and sometimes contradictory notions of corruption, some of which will be addressed below. There is not simply one evolving discourse on corruption where there was a gradual transformation in its meaning over time. Rather, despite the prominence of official definitions, mainly stemming from the OECD Anti-Bribery Convention and the related work of TI, we uncovered several competing and at times overlapping meanings which pose different questions about what the ‘problem’ is and its possible solutions. Due to space constraints, we focus in this paper on the second major insight. We identify three phases in which this corruption rhetoric emerges. These phases do not unfold in a chronological or conceptually linear manner (i.e., they are not necessarily linked events that build from each other) but instead mark notable moments in which struggles over the meaning of corruption play out in the news media. We conclude that what is often depicted as a check on power – a free press – is complicit in the rise of an anti-corruption rhetoric that has done more to justify the expansion of corporate power than contribute to the reduction of the corrosion and distortion of public processes and institutions by private interests. Overall, Canadian newspapers have reproduced an uncritical and incoherent Canadian popular discourse on corruption.

## Rationale and Methodological Moorings

Our methodological mooring is in an abductive materialist analysis that takes our empirical material to be what Alvesson and Kärreman (2011, pp. 12–15) call a *critical dialogue partner,* which entails moving “…beyond a grounded theory-like codification and the discovery of categories” to critically engage with the empirical material to “...challenge, refute, refine and illustrate theory.” Our objective is to use the news items to help generate concepts to better understand the (anti)corruption problem, rather than to marshal our materials as data against which to test hypotheses or from which to offer inductively derived descriptions. In this sense our empirical material is “a resource for developing theoretical ideas *through the active mobilization and problematization of existing frameworks”* (Alvesson & Kärreman, 2011, p. 4, emphasis added).

This strategy requires creative and active use of a range of conceptual tools and iterative engagement with empirical materials rather than one dominant conceptual framing (deductive) or a descriptive grounded theory approach (inductive). An abductive materialist approach enables ‘seeing’ from multiple perspectives, drawing from our “theoretical repertoire” to provide “illumination, insight, and understanding” and to “generate new conceptualizations” (Frauley, 2021, p. 9). This is not a static or fixed process, but instead an “iterative interplay between generating ideas and engaging with empirical materials” (Frauley, 2021, p. 9).^2^

We draw on several theoretical resources furnished by social constructionism and political-economy as well as insight from the critical literature on anti-corruption(ism). Of note is Hall et al.’s (2017) classic analysis of a mugging panic in Britain in the early 1970s. They argue that institutional rules and pressures on news media to meet tight deadlines and to be seen as impartial and objective (through the use of seemingly reliable, credible, and detached experts), “serve powerfully to orientate the media in the ‘definitions of social reality’ which their ‘accredited sources’ – the institutional spokesmen – provide” (Hall et al., 2017, p. 58). These “primary definers” – motivated insiders and stakeholders – help to “establish the initial definition or primary interpretation of the topic in question” (2017, p. 61). Important for our purposes is Hall et al.’s (2017) argument that news media are not primary definers in this process, but instead a conduit through which powerful voices are heard and reproduced. The primary definers populating Canadian news on corruption and anti-corruption include representatives of the OECD, TI, and the WB, among others, as well as government bureaucrats and professionals working in the business sector. As Hall et al. (2017, p. 60) note, because news output would be near impossible without participation of industry spokespeople, “… the media come, in fact, in the ‘last instance’, to reproduce the definitions of the powerful, without being, in a simple sense, in their pay.”

Herman and Chomsky’s (1988) “propaganda model” of mass media is also instructive. They speak to structurally embedded “filters” within the mass media that condition and shape the raw material and determine newsworthiness, leading to alignment of news with hegemonic political and economic interests. These filters through which the powerful are able to fix the premise of a discourse are: *ownership* (concentrated and profit-seeking corporations); *funding* (corporate advertising which commoditizes readers/viewers); *official sources* (powerful and privileged spokespeople whose organizations spend vast sums on public relations and lobbying); *flak* (blowback generated by financially or politically privileged actors against unfavourable media coverage); and *othering* (identifying *an enemy* to be marginalized). Official sources or primary definers are significant for our analysis in that they both fund media and provide the raw material for news commodities (Herman & Chomsky, 1988, p. 18). Flak, in our case its avoidance, works alongside needed access to official sources to suggest why news media in our study adopt uncritically rather than scrutinise the language of their official sources. This leads to media complicity with the claims of powerful interests rather than to a check on or scrutiny thereof. Othering constructs an enemy around which a consensus can be formed: in our case that corruption as bribery is an individual transaction by ‘bad apples’ that is harmful to democracy, robs the political system of its capacity to aid the poor and vulnerable, and hurts economic prosperity.

Accounting for the broader conditions within which news media operates is also necessary for understanding processes of ideological reproduction. The global alignment of liberalized market economies emerging from the stagflation of the 1980s – generally referred to as the neoliberal and post-social era (O’Malley, 1996; Sears, 1999; Teeple, 1995) – forms the backdrop to our enquiry of newspaper coverage of the global anti-corruption movement. This new phase of global capitalism brought with it not only global market alignment and the enormous expansion of corporate power, but also the increased importance and authority of global transnational governance institutions such as the WB, IMF, and OECD. These international institutions set the rules and norms for the economic and political coordination of member states, which impacts each member’s domestic and foreign policy. The OECD is of paramount importance for G7 policy coordination, including alignment of anti-corruption laws, enforcement, and publicized rhetoric (Katzarova, 2019; Rose, 2015).

Katzarova (2019) argues the evolution of a global consensus around corruption is the outcome of a protracted consensus-building exercise by the United States (US) across international and regional governance bodies to internationalize its *Foreign Corrupt Practices Act* (FCPA, 1977). From the mid-1970s onward this involved the US pushing to define corruption as bribery and to criminalize it. Initial efforts at the UN level in the 1970s failed when member countries from the Global South preferred a definition of corruption centred on the undue influence of corporate power in political matters. Subsequent efforts by the US through the OECD in the 1990s were more successful in cementing the notion of corruption as bribery. By then, the bribery rhetoric aligned well with prevailing neoliberal ideals that downplayed state-centred regulation in favour of individualistic notions of corruption and private sector self-regulation. Katzarova (2019, p. 32) usefully identifies three stages of development of corruption as a global governance problem. *Phase 1*, from the early 1970s to the mid 1990s, concerns the early years during which corruption was not seen as a pressing international problem and when the US failed to advance its corruption as bribery framework. *Phase 2,* from 1994 to 2005, “marked the institutionalization of the issue” entailing “a plethora of formal international legal arrangements, a boom of codes of conduct and vast media coverage” which elevated corruption “to the top of the international public, media and policy agenda” (Katzarova, 2019, p. 32). *Phase 3* began around 2005 and represents a “new [and ongoing] phase of normalization.”

The anti-corruption movement, emerging in the mid 1990s and led by the World Bank (Sampson, 2010, p. 273), is characterized by Engels (2018, p. 179) as the “third phase” in the long-running debate about corruption. This third phase, he (Engels 2018, p. 180) argues, signals a “compliance revolution” entailing the exponential increase and broadening out of anti-corruption legislation in capitalist democracies and beyond. With this we have witnessed a political turn by the WB and IMF as they began imposing neoliberal market-friendly regulation on the states that became their “clients” to radically alter those states’ economic and political institutions, aligning these with global market capitalism (Mackenzie, 2006; Wedel, 2012). In cases where client states were already ‘developed’ but perhaps economically wayward (such as Greece) or emerging from the former Soviet Union (such as Ukraine), re-alignment was also imposed (Brown and Cloke, 2004; Hindess, 2005). However, there is now a growing chorus of scholars who question the impact and effectiveness of anti-corruption laws in curbing corruption (Andersson and Heywood 2008; Heywood, 2018; Hindess, 2012; McVittie and Sambaraju, 2019; Sampson, 2015). The seemingly lack-lustre results of the anti-corruption movement might be understood with reference to the dominant view held by a vast number of scholars who see corruption as bribery and therefore as what is known as “bureaucratic corruption”. This mainly, but not exclusively, entails adopting a principal-agent understanding that reduces corruption to greed. This view negates the impact that powerful institutions can have on shaping anti-corruption practice. The focus on bureaucratic corruption “displaces any serious effort to address the question of political corruption as a matter of private interests within the policy-making process” (Bratsis, 2014, p. 110). Political corruption involves decision making at the very heights of power “very removed from the lives of common citizens” (Bratsis, 2014, p. 110).

Building from Katzarova’s three stages of development of the corruption ‘problem’, along with insights from Hall et al., Herman and Chomsky, and the literature critical of the anti-corruption movement, we sought to understand our news items as “texts [which] are oriented towards creating effects, rather than mirroring a phenomenon in social reality” (Alvesson & Kärreman, 2011, p. 29). That is, we wanted to examine how news items on (anti)corruption *reproduce* the voices of the powerful rather than merely report on a problem, and therein reflect an anti-corruption politics occurring at a broader, global level. In so doing, we are able to examine newsmaking as one element of the political corruption process, as it operates as a mechanism or conduit to channel the voices of powerful actors dominating at the international level in the conflict over the formulation of the corruption problem. We are not aware of any other scholarship that utilizes this approach in the manner we do to understand the role of newsmedia as a mechanism that steers popular and political-legal notions of corruption toward bureaucratic corruption and away from any scrutiny of political corruption.

The next section identifies three phases in Canadian newsprint reporting on corruption and anti-corruption. The first phase, which we dub the *shallow* or *parochial* phase, from the 1980s to early 1990s, contains reporting depicting corruption largely as a problem of the ‘other’, whether corrupt individuals (i.e., bad apples), underdeveloped or wayward European states, or authoritarian states (such as the Soviet Union) with corrupt state-run enterprises. Phase two, the *importation* phase from the mid 1990s to the early 2000s, involves Canadian newspapers relying almost exclusively on the rhetoric of primary definers (business leaders, transnational governance institutions and TI) within the burgeoning anti-corruption movement. The final, *shambolic* phase, from the early 2000s onward, witnesses uncritical reporting of the corruption as bribery discourse established during the importation phase, along with new and fragmented ways in which various conceptualizations of corruption expand into different realms. Overall, what we find most striking about Canadian newspaper treatment of corruption during these phases is that what is often depicted as a check on power – that is, a free press – is complicit in the expansion of corporate power in reproducing the language and positions of primary definers.

## The Shallow or Parochial Phase

The first phase is the shallow or parochial phase and includes news items from 1980 to the mid-1990s. The depth of engagement of newspaper coverage of corruption during this period lacks a central focus and mostly casts aspersions at morally corrupt individuals or governments. Reporting on corruption within Canada tends to centre on corrupt individuals (e.g., morally bankrupt politicians), whereas reporting on corruption elsewhere references ‘inherently’ corrupt systems, governments, and cultures. There are two main categories of newspaper reporting during this phase: (1) items that equate corruption with bribery in “Third World” or “foreign” countries with “underdeveloped” democracies and industries dominated by state-controlled enterprises; and (2) items that use corruption in pejorative terms to describe politicians who succumb to lobbying efforts or corporate donations. Together these items contribute to a highly individualized conceptualization of corruption (i.e., a personal moral failing) and hold it to be a problem of ‘other’ countries.

When it comes to reporting of corruption outside of Canada, a cultural notion of the term emerges that centres on national or regional norms and values and is commonly applied to “Third World” or developing countries by emphasizing their governing ideologies. The general assumption is that corruption is the natural outcome of socialist governments, state-owned/operated enterprises, and weak democracies. Almost without fail, these stories report on the steps being taken (if any) by governments to ameliorate their countries’ political and economic conditions, which for journalists and their sources boiled down to how effectively governments had liberalized, privatized, and democratized.

A series of items on corruption in African countries are instructive in this regard. One item decries Tanzanian President Julius Nyerere’s “African socialism” for its “state-controlled corporations” that increased the “exploitation of his citizens”. As the author argues of the President’s socialist bent, “Unwilling to admit that many state trading corporations are unviable, he has taxed his peasants to make up for their losses and corruption.” The item notes overlooked benefits of “private business” for generating “economic value” (Winsor, 1981a). A related item characterizes the state-run foodgrain company, National Milling Corporation, as fraught by “high prices, shortages, and corruption” (Winsor, 1981b). Likewise, writing about the situation in Tanzania, one author argues that “under the economic control of a morass of 400 parastatals (Crown corporations), nationalized agricultural production has declined, state factories have failed, food prices and food distribution have become chaotic and mismanagement and corruption have become endemic” (Valpy, 1985).

In each case, corruption is self-evidently explained by the fact that commerce is state-run, with some form of privatization as the logical corrective. In the case of “Tanzanian socialism” the government’s acknowledgement of its errors in economic nationalization and of the appropriate role of private enterprise was held to signal some promise for the country’s future under new leadership.

The Government today has acknowledged that it was wrong to nationalize sisal production, an important export crop where production under state management declined 40 per cent in five years. … The Government now allows private enterprises to exist in peace when they do better than competing state enterprises, instead of continuing to try to force them out of business (Valpy, 1985).

As a Globe and Mail item notes of the situation in Nigeria, although President Babangida “moved to privatize the state-owned corporations and stamp out corruption,” the country’s “fundamental problems” remained unresolved as “Western diplomats report that the ‘levels of corruption are staggering’” (Van Niekerk, 1993a). Another item describes Zaire - whose “economy suffered corruption on a scale rivalled only by Nigeria” - as an exemplar of “economic rectitude,” taking a positive turn towards meeting IMF loan conditions. Having “presided over an economic disaster which began with an ill-conceived nationalization program,” President Mobutu’s recent reforms were held to position the country as an emerging “model IMF client,” citing steps the government had taken to “strengthen the management of the state-owned copper and cobalt mining corporation,” and to begin replacing an “allegedly corrupt state mineral marketing corporation” (Holman, 1984).

A similar theme emerges from newspaper items on Asian economies. An editorial on the election of Corazon Aquino as president of the Philippines outlines “the enormity of [her] task of righting a badly wronged economy” after 20 years under the leadership of Ferdinand Marcos and his “cronies” (Globe and Mail, 1986). Among the tasks was “breaking up the corrupt and inefficient monopolies that control the country’s important sugar, coconut and grain industries.” With the US “prepared to step in with a mini-Marshall Plan to help boost the Philippine economy, and an improved political climate” after Aquino’s election, foreign investment was depicted as the solution. Particularly hopeful, according to the editorial, was the fact that Filipinos were “[a] well-educated and Western-oriented population, willing to work hard for wages that are a fraction of those paid in Singapore and South Korea.” Economic development needed to be rapid, for “[a] vicious, Communist-oriented insurgency movement lies waiting for the people to grow disenchanted once again” (Globe and Mail, 1986). By contrast, the 1981 election of San Yu as president of Burma was less encouraging given he was “expected to follow the same Socialist path as his predecessor” that had had “disastrous results” (Reuter News Agency, 1981). Nationalization of the “resource-rich economy” had failed after twenty-two previous government corporations “became known for corruption and inefficiency,” eventually spawning “a country-wide black market that still thrives” (Reuter News Agency, 1981). In a similar vein, state corporations were singled out for rendering China “rotten with corruption”:

State corporations and plum political positions are riddled with the offspring of political bigshots, whether competent or otherwise. The system itself works on the basis of friendship, payoffs and personal favors. The introduction of economic reforms has only aggravated the situation, as there is now more money around, and more pressure to get a piece of it while it lasts (McParland, 1992).

Countries engaged in market liberalisation were less likely to be seen as corrupt (or at least as motivated to shed their corrupt ways). By the early 1990s, Latin America had “taken major steps toward economic restructuring and democratic government” (Toronto Star, 1992). The region’s troubles were held to be a combination of issues experienced in different parts of the “Third World”. Like African and Asian countries, Latin America was afflicted with rural poverty and perpetual “warlord violence”, and like post-Soviet countries, it had “a crumbling infrastructure and a corrupt, governing bureaucracy getting rich on its connections with big Western corporations” (Toronto Star, 1992). Meanwhile, Mexico was the posterchild of the region’s economic development. With his “sweeping plans to modernize Mexican society,” President Carlos Salinas de Gortari was praised for having “reversed the past policy of heavy government involvement in the economy, privatized many state corporations, waged war on corruption, cracked down on the drug trade, developed new social programs and strengthened both democracy and the rule of law” (Crane, 1990).

After a century as the sleepy land of tequila and sombreros, Mexico is rapidly leaving behind the Third World in a bid to become North America’s new industrial powerhouse. … The linchpin in Salinas’s dramatic quest is free trade with the United States and Canada, an idea he unexpectedly sprung on his continental neighbors in 1990. By taking on everything from state-owned corporate behemoths to bribe-demanding customs officials, Salinas has won tremendous respect from a Mexican population fed up with corruption and do-nothing government (Whittington, 1992).

Any reference during this phase to Canadian companies being involved in corruption abroad typically characterized them as being exposed to, or victims of corruption when conducting business in countries like Mexico, China, or Nigeria. Ultimately, however, the message was that companies should not be deterred from operating in other countries, underpinned by claims that dealing with corruption was an increasing priority and that free trade was a vital part of these developments. An item on corruption in Nigeria cites the “gullibility” of “Western business people” as a source of corruption. Citing Nigerian government sources, the story notes how foreign companies fall into corruption “traps” because they are so focused on the bottom line. However, despite these trappings, the story also cites a Canadian official who lauds the trade opportunities: “There’s a lot of bona fide business here for foreign companies who want to do it right” (Van Niekerk, 1993b). Another item documents the difficulties the Canadian extractive industry – itself a source of much controversy – faces when conducting business in certain regions. As the item notes,

Most of the foreign properties run by Canadian companies are in Mexico, Venezuela, Chile, Peru and Argentina – countries with relatively well-established political and judicial systems. But a growing number are in less stable regions where justice is dished out at the whim of dictators, corporate law is still a work in progress and corruption is rampant (Howlett & Drohan, 1997).

In comparison, the relatively minimal reporting on corruption in the Canadian context more narrowly construes it as individual cases of wrongdoing (the corrupt ‘bad apple’). Individualized accounts included stories about Canadian businessman, John Doyle, who was accused of defrauding Javelin, the mining company he founded (Gibb-Clark, 1982). One story involved a reporter interviewing Doyle at his home in Panama, where Doyle had fled to avoid prosecution for his crimes, and which provides ample opportunity for him to deny his fraud charges (Freeman, 1985). The item focuses mainly on Doyle’s personality, as a corrupt character, which departs from the reporting regarding corruption in African countries where the focus is more about the inherently corrupt nature of governments and economies. In further contrast with reporting on corruption in ‘other’ countries, news items concerning corruption in Canadian politics was more ambivalent, depicting it as a problem of bad perception caused by corporate donations to political parties (e.g., Corcoran, 1993; Johnson, 1980; Winsor, 1991). Similarly individualized references to corporate corruption emerge in reporting on a crisis in public confidence in business following a series of corporate scandals, such as the Dalkon Shield, fraud on Wall Street and in the Chicago Futures Exchange, and the Union Carbide disaster in Bhopal (Reid, 1989). The primary focus here is waning consumer confidence when “corrupt cultures” go sour or when there is a lack of “integrity” amongst some individuals within a company. As we shall see, this notion that ‘true’ corruption is something that happens elsewhere – to ‘them’, in ‘under-developed’ countries – and what happens to us is about bad apples or bad corporate cultures, becomes a prominent theme in the next phase of news items.

## The Importation Phase

While remnants of shallow conceptions of corporate corruption continue through the late 1990s, Canadian newspapers during this time move into a second phase of reporting – the importation phase – in which the focus and driver becomes the global rise of the anti-corruption movement, which coalesces around an emerging narrow consensus of corruption as bribery. In the process, Canadian print media uncritically adopt the language given to it by external experts such as those from the US, OECD, WB, and TI. The impetus for this second phase is the advent of the 1996 OECD Anti-Bribery Convention and the concomitant emergence of an anti-corruption narrative in the political sphere, prompting journalists and other commentators to pontificate over the agreement and strategies for its national implementation and enforcement.

Four themes emerge from the news items during this phase: (1) growing concerns with bribery undermining fair competition in ‘free’ markets; (2) claims about the victimization of corporations operating in those markets; (3) calls to “fight” corruption as remedy for under-developed and undemocratic countries where bribery prevails and for governments and corporations in the Global North to promote their brand of good governance; and (4) reporting on TI, a think-tank and anti-corruption advocacy group started by a former WB executive. TI emerges as the prominent corruption expert and begins popularising its agenda and rhetoric on the topic, generating a common language for news outlets to frame debate and discussion of corruption as a state and corporate phenomenon. TI’s Corruption Perception Index (CPI) becomes a standard source for journalists to report on the corruption ‘problem’ during this period.

Reporting on corruption during the mid-to late-1990s begins to raise the spectre of the corruption of global markets, led primarily by US interest in forging a level economic playing field in which all participants would be subject to the same rules and conditions set out by the OECD Anti-Bribery Convention. As the only country that had banned foreign bribery, the US and its corporations were depicted as unfairly punished in the global economy, as other jurisdictions had not followed their lead. In effect, US law rendered corruption a competitive advantage for those competing with US corporations for market share.

One could well imagine that corporations from countries outside the United States were grateful for the moral strait jacket the Americans had electd [sic] to wear. For years, the lack of restrictions on, and even tax deductibility of, their bribery seemingly provided non-U.S. companies with a competitive edge (Johnston, 1998).

While the [FCPA] may have eased American consciences, it has been costly. According to the U.S. Department of Commerce, American firms lost 100 foreign contracts worth $45 billion to overseas rivals between 1994 and 1995 because they were cut out of the circle of graft and corruption (Handelman, 1996).

Further depicting global economic conditions as unfair to the US, legal variation between states was invoked to make sense of the US Security and Exchange Commission’s (SEC’s) finding, shortly before the OECD Convention’s signing, “that U.S. corporate bribery of foreign officials may be surfacing as a significant problem for the first time in 20 years” (Bloomberg Business News, 1997). Without the rest of the world following the US lead, US corporations were forced to breach domestic law in order to compete in foreign markets despite “significant progress for Washington in its campaign to deal with international corruption” (Oxford Analytica, 1996) and “sustained diplomatic pressure” (Mason & de Jonquieres, 1997) to obtain “a level playing field … since its law was passed” (Handelman, 1996).

As part of US concerns about unfairness caused by legal variation, corruption was also framed as a global economic governance issue that negatively affected all countries. Corruption was thought to be “the rot at the heart of the new international economy” that was “rapidly taking on crisis proportions” (Handelman, 1996). In keeping with the cultural conception of corruption as a foreign phenomenon, transnational corporations and the Western governments meant to regulate their operations abroad were slowly coming under scrutiny, or so it seemed:

A very large part of the blame rests with multinational corporations who are headquartered in the West. Before anyone in the West gets on a high horse and starts talking about corruption in developing countries, they need first of all to ensure they have laws in place that represent a strong disincentive to multinational companies to give bribes in foreign business (Globe and Mail, 1997).

In a story on TI founder Peter Eigen’s visit to Toronto to promote the organization’s Canadian chapter, he explained that “corruption also harms developed countries like Canada because it undermines corporate integrity and ethics” (Dalglish, 1996). Abiding by foreign political and economic norms was held to have a “boomerang effect” whereby paying bribes “builds pools of illicit offshore capital that can eventually be used against the best interests of developed nations” (Dalglish, 1996). As Eigen implied in his anti-corruption advocacy, any developed country without legislation like the US was at risk of Watergate-like scandals. In this way, corporate corruption is imported to Canada as a domestic regulatory issue with potentially significant consequences for Canadian democracy and markets.

In response to concerns with bribery’s impact on ‘fair’ competition comes demands to ‘fight’ corruption. As one opinion item argues, “Canada and other nations should continually reaffirm the message that bribery and corruption – too often dismissed as merely the cost of doing business – will no longer be tolerated” (National Post, 1996). “Canada urged to take tougher stand on Third World Corruption” claims another item, because corruption “undermines corporate integrity and ethics” (Dalglish, 1996). In addition to reporting on the OECD’s Anti-Bribery Convention, various stories also position countries and companies in the Global North as leaders in good governance and social responsibility. It is here that anti-corruption, with support of academic knowledge claims, becomes intertwined with the idea that companies need to ‘do the right thing’. Legal scholar Errol Mendes, for instance, penned an opinion piece in which he advocates for governments and corporations to endorse “…a set of ethical principles companies will seek to abide by in their international business operations” (Mendes, 1997). Other items also raise the link between combatting corruption and good corporate citizenship. As one item notes of the problem of corruption in Nigeria:

We understand the preference of Shell and other companies to concentrate on business and avoid politics. But good corporate citizenship extends far beyond the bottom line. By turning a blind eye to corruption and tyranny in Nigeria, these multinational companies have missed the chance to play a constructive role in Nigeria’s painfully slow evolution toward democratic rule (Hamilton Spectator, 1998).

There is also frequent reference during this phase to TI and its CPI. This reporting provides a potent framework for the corruption ‘problem’ in that it generates a steady diet of reporting on the topic that redirects concern about corruption away from countries in the Global North to so-called underdeveloped, undemocratic nations. “Canada is ranked as the most squeaky clean of the Group of Seven industrialized nations in an international survey of corruption in 54 countries”, is typical of the uncritical reporting of the CPI (Morton, 1996). There is virtually no reporting that is critical of the CPI despite its reliance on the ‘perceptions’ of mostly corporate leaders from the Global North. One exception is an item in which a representative from TI responds to questions about the survey’s bias by admitting there was a tendency for “Western” perceptions to dominate. At the same time, however, the representative argues that, while companies from the West are not immune to corruption, “[c]learly in the poorer countries with more fragile political institutions, the impact of corruption tends to be far greater and far more burdensome on the poor.” The same representative also dismisses as “nonsense” any suggestion that “capitalism increases corruption” (Globe and Mail, 1997).

## The Shambolic Phase

With the narrow and legalistic conceptualization of corruption emerging from the importation phase, along with continued usage of parochial meanings of the term, the next phase of reporting enters a shambolic phase beginning in the early 2000s. By this we mean that the corruption rhetoric loses any sense of discursive order as the term’s meaning is further fragmented and anti-corruption measures are paradoxically construed among the primary definers within the anti-corruption movement. To document this phase, we focus on two exemplary events that dominated news coverage during this period: the wave of corporate scandals famously involving the Enron collapse, and, in Canada, the SNC-Lavalin foreign bribery scandal and its domestic political fallout. The shambolic rhetoric manifests itself in three major themes: (1) extending the use of the term corruption to phenomena beyond its conventional scope; (2) declining support for anti-corruption measures as anti-capitalist; and (3) increased resistance towards state intervention of corporate activity in favour of strategies of self-regulation, including corporate social responsibility, deferred prosecution agreements, and reforming corporate culture.

Corporate scandals at Enron, WorldCom and other major U.S. firms brought with them a crisis of confidence in Western markets that required immediate attention (Lauria, 2002; Toronto Star, 2002). Reporting reveals the popular framing of these scandals as eroding “public confidence in corporate America” (Gordon, 2002), while President Bush’s “use [of] the power of his office to root out corporate fraud” made it “clear that American capitalism is in deep trouble” (Toronto Star, 2002; see also Corcoran, 2002). Confidence in Canadian business was also at issue, as Prime Minister Chretien’s “loose economic lips” revealed that “his government is ‘very worried’ and ‘afraid’ about the damage the U.S. corporate scandals could inflict on the Canadian economy” (Globe and Mail, 2002; see also Scoffield, 2002; Morton & Fife, 2002). While these scandals were not orthodox examples of corruption in that they more closely resembled classic corporate frauds or pump-and-dump schemes, the burgeoning corruption rhetoric provided a convenient way to frame the problem in individualistic terms (i.e., bad apples or corporate cultures gone awry) that ultimately called for improved ethics and social responsibility.

The term corruption was used in news items to describe the individuals involved in fraudulent accounting practices and the corporate cultures they had cultivated as executive leaders. The wave of corporate scandals was understood as the manifestation of a “culture of greed” that rewarded people “who lack strong moral foundations” (Pitts, 2002). Motivated by greed, according to one writer, “Enron was spurred by a corporate ‘culture of corruption’ in impoverishing the state” (Olive, 2002). The emergence of “a new class of piratic manipulators … with the intent of enriching themselves at the expense of consumers” (Olive, 2002) was not, according to U.S. Federal Reserve chairman Alan Greenspan, indicative of “humans … becom[ing] any more greedy than in generations past,” but rather “[a]n infectious greed … grip[ping] much of our business community” in a regulatory environment with more “avenues to express greed” (Morton & Fife, 2002). Focusing on the “rogues” by harshening punishment and increasing enforcement was widely held to miss the fact that “the problem is endemic to corporate culture” (The Portfolio Doctor, 2002). Instead, responsibility to shareholders needed to be incentivized by reordering corporate priorities: “It may well be that most people are corruptible, given the right circumstances. But we also believe that most of those who are ultimately corrupted lack, right from the beginning, the very qualities we shareholders need in our leaders” (The Portfolio Doctor, 2002).

The Enron-era scandals led to demands for stricter laws to combat corporate greed and to punish malign corporate actors. The US government led the way in 2002 by introducing the Sarbanes-Oxley Act aimed at improving financial transparency and reporting within companies. The Canadian government quickly followed suit with the introduction of markets fraud legislation in 2004. While we do not have the space here to detail the struggles to introduce and enforce these laws (see Snider, 2009; Soederberg, 2010), our news items generally cohered with what commonly happens in the wake of efforts to criminalize the powerful. That is, the “cultural permission” to punish corporations and corporate executives (Snider, 2009) was soon overtaken by concerns that criminal laws were too harsh – that they were unnecessarily impeding corporations from competing in much vaunted global markets – and that it was more appropriate to seek relief through improved corporate governance and social responsibility measures (read: more self-regulation). An editorial impugning the “new federal charge against alleged corporate ethical breaches known as Corporate Fraud” is illustrative:

Corporate Fraud is a mythical Trojan beast imported into Canada from the United States by regulators and politicians looking for fresh opportunities to be seen to be doing something about something. … To justify this new spending and the new penalties, the ministers trotted out the usual bogus hocus pocus of modern regulation. Mere mention of the words Enron and Worldcom is a licence to unleash new regulatory dogs, even if the reference lacks logic or relevance (Corcoran, 2003).

Regulatory reform was soon portrayed less as a check on corrupt corporations and more as an assault on capitalism itself. One writer, relying on corporate lawyers’ views on the $25-million penalty imposed on Lucent Technologies for its failure to co-operate with the SEC, reported on the “growing concern[] that basic rights are being trampled in the effort to stamp out corporate corruption” (Maich, 2004). Corporations were depicted as defenseless against an overpowerful SEC:

After the slew of corporate scandals exposed over the past few years, a sweeping clean up was clearly in order. But with the SEC cracking down on any and all opposition, critics now wonder if the government is taking away a company’s right to self-defense” (Maich, 2004).

Another item declared Sarbanes-Oxley “usher[ed] in the greatest-ever government assault on business and private enterprise” (Corcoran, 2004). Thought to reflect the anti-capitalist leanings of some politicians and news media, the suggestion was that it

… was only part of the anti-business campaign that has swamped U.S. and Canadian corporations over the last two years. The [Eliot] Spitzer extortion rackets against investment dealers and mutual funds, over offences and practices that were minor or even legal, has further undermined business activity. Billions of dollars have been diverted to fighting regulators and paying fines that serve no investor purpose (Corcoran, 2004).

Within this context of increased hostility towards state intervention, news items reported on various efforts to confront corruption by, for instance, addressing executive compensation and improving business ethics. When it came to executive compensation, the message was that steps were needed to rein in greedy executives. As a Toronto Star story noted, the “systemic risk of corruption” could be reduced by requiring

… the expensing of stock options so shareholders can see their true cost. Another is to allow senior executives to exercise stock options but not sell their shares until a cooling-off period after they have left the enterprise. A third is to make mandatory at company annual meetings a vote on executive compensation (Crane, 2002).

One item recommended executive compensation committees (Anderson, 2002), while another suggested a new executive compensation system that ensures “only those who’ve really earned it will be highly paid” (Wilson, 2002).

With executive reputation in tatters, the rhetoric shifted to rescuing those charged with running corporations. Business schools and ethics figure prominently in news reporting on anti-corruption efforts post-Enron. Carleton University’s Sprott School of Business, for instance, introduced a certificate program in ethics and values, based on “the argument that rules and regulations established to guide ethical behaviour only encourage people to break them, and that this is nowhere truer than in the U.S., where greed and corruption have overwhelmed the better judgement of prominent corporate leaders” (Morrissy, 2002). Similar stories emerge about the creation of ethics classes in MBA programs at Harvard and Stanford (National Post, 2005), or that improved ethics and leadership training would help business schools to “prevent more of their grads from becoming the stuff of sordid headlines…” (Sachdev, 2003). Another item laments how elite business schools erroneously emphasized “the art and craft of making money but not … ethical stewardship of shareholders’ money” (Pitts, 2002).

Not only did the corruption rhetoric extend to corporate accounting fraud to further fragment the term’s meaning, its narrow political-legal conceptualization fell into disfavour in relation to a classic case of foreign bribery perpetrated by global engineering giant SNC-Lavalin. In this major Canadian case where the *Corruption of Foreign Public Officials Act* (CFPOA, 1998) clearly applied, the primary definers shift away from anti-corruption measures as taking a tough stance against corporations in favour of a compliance model that emphasizes corporate culture reformation and punishing individual wrongdoers instead of innocent employees and other bystanders. This is reflected in the dominance in coverage about the need for deferred prosecution agreements (DPAs), referred to as Remediation Agreements (RAs) in Canada.

Soon after it was reported that former SNC-Lavalin executive Riadh Ben Aissa was arrested in Switzerland on accusations of bribing the Gaddafi regime in Libya, news items began following the measures taken by the firm to reform its corporate culture. At the risk of “signal[ling] just how deep SNC’s board believes its internal ethics problems run,” the company hired Watergate investigator Michael Hershman as an independent compliance advisor (Van Praet, 2013a). Although “[t]here is no evidence to date that SNC-Lavalin’s corporate culture is as broken as that of Siemens,” Hershman’s experience revamping the latter’s compliance controls to ultimately settle its bribery charges was sought by SNC-Lavalin in its pursuit of a similar outcome (Van Praet, 2013a). Four months later, the newly created chief compliance officer (CCO) position was filled by former Siemens CCO Andreas Pohlmann to establish “a new business ethos at SNC” by implementing a “new compliance program” (Cousineau, 2013). Despite the incomparable corporate cultures at SNC-Lavalin and Siemens, “[i]t is SNC’s culture that Mr. Pohlmann is out to reshape” (Cousineau, 2013). Within a month of his hiring, Pohlmann followed a similar strategy he used at Siemens by offering an amnesty program to employees, “the first such move in corporate Canada” (Van Praet, 2013b). The novelty of the amnesty program to Canada is an important detail in that it foreshadows the next issue that arises in SNC-Lavalin-related news items: the absence of deferred prosecution agreements (DPAs) in Canada.

The SNC-Lavalin scandal entered the political realm in 2019 when the media reported that Prime Minister (PM) Justin Trudeau and some members of his inner circle had attempted to strong-arm then Attorney General (AG), Jody Wilson-Raybould, to overrule the Director of Public Prosecutions (DPP), the head of the independent Public Prosecution Service of Canada (PPSC), who had decided to proceed with the prosecution of SNC on charges of foreign corruption and fraud. The PM’s preference was for prosecutors to negotiate with SNC on a RA, which his government had added to the *Criminal Code* in 2018. A successfully negotiated RA would have allowed SNC to avoid a criminal conviction by meeting several conditions, including an admission of responsibility, a fine and other penalties, cooperation with authorities on related investigations, and creation of internal policies and measures to prevent future offending (Bittle & Quaid, 2022). In addition to laying bare the symbiotic relationship between states and corporations (Tombs & Whyte, 2015) – namely that states are in a contradictory position potentially punishing the very organizations they helped create and which they rely on politically and economically – the ensuing debate about how best to respond to this case generated rhetoric about the need to avoid being overly harsh by embracing RAs to provide companies with an opportunity to root out bad apples and reform corrupt corporate cultures.

Preceding the introduction of RAs, news items uncritically reported on SNC-Lavalin’s efforts to lobby the federal government to introduce this measure (e.g., Adams, 2017; Van Praet, 2018). Without a DPA regime like in other countries, Canada was “behind the ball” on anti-corruption according to a lawyer specializing in anti-corruption law (Van Praet, 2018). As others lawyers suggested, a gap in Canada’s enforcement of white-collar crime could be filled by a DPA regime (quoted in Van Praet, 2017; Aylward, 2018). Furthermore, TI Canada published a report “urging the Canadian government to adopt a DPA mechanism modelled closely on the British system. The group said any scheme should include financial reparations, sincere compliance reform and accountability of individual wrongdoers” (Van Praet, 2017).

Further justification for DPAs surfaced in relation to the perceived economic damage that companies and their employees would face in the wake of a criminal prosecution. As one item noted: “The government says DPAs may mitigate unintended consequences of a criminal conviction for blameless employees, customers, pensioners, suppliers and investors. Convictions could result in job losses and wider negative economic implications” (Marowits, 2017). Similarly, by claiming that the DPA debate was about Canada’s best interests, SNC-Lavalin CEO Neil Bruce intimated “that going after SNC itself, as opposed to the individual sleazoids responsible for the corruption, would risk 9000 Canadian jobs and perhaps even the Montreal head office, if the sagging share price were to lure bargain-hunting takeover artists” (Reguly, 2018). As one headline read, “Are the right people being punished at SNC-Lavalin? The executives responsible for the corporate scandal should be brought to justice. But we shouldn’t punish those trying to save the firm” (Hood, 2018). “Corruption should not be tolerated,” one writer suggests, “[b]ut the fight against it would be better served by increasing penalties for the individuals responsible” (Watson, 2018). The mere fact that Canada did not have a DPA regime at the time was seen as a competitive disadvantage: “Chief executive Neil Bruce has said the company lost two foreign contracts because competitors used these agreements” (Marowits, 2017). Overall, given the rarity of corruption charges in Canada, their use against SNC-Lavalin raised concerns among political and business leaders in Quebec, leading to SNC-Lavalin’s depiction as a victim (Van Praet, 2015).

Despite the enactment of a RA regime, there were nevertheless differences of opinions amongst primary definers regarding the use of criminal prosecutions. However, these conflicting perceptions of Canada’s anti-corruption strategy only serve to capture the shambolic character of the corruption rhetoric. On the one hand, domestic actors challenged Canada’s approach as “overkill” (Corcoran, 2014; Corcoran, 2015; see also above). On the other hand, international actors praised Canada’s increased enforcement of the CFPOA: “Canada’s standing on the international stage got a boost Monday, because of the RCMP’s recent crackdown on bribery cases – including the hunt for alleged corruption at SNC-Lavalin Group Inc. The [OECD] released an updated report card on Canada’s anti-bribery campaign, and credited Canada for charging individuals at SNC-Lavalin…” (Cousineau & Gray, 2013; see also Tedesco, 2014; Perreaux et al., 2015). Regardless, criminal law was seen as a tool of last resort, with dominant rhetoric favouring strategies centred on improved corporate ethics and social responsibility.

## Conclusion

Our analysis of Canadian print media coverage of corporate corruption reveals a story about the entrenchment of corporate power that is linked to development and investment opportunities in new or expanding markets (e.g., new markets via fall of former communist countries or expansion of free trade globally) which is often missing from the anti-corruption literature. This story unfolds over a forty-year period in which International Financial Institutions (IFIs) such as the WB and IMF, along with governments of the Global North and the OECD, engaged in various free-trade efforts to refashion the global economy in ways that provides relatively stable and predictable markets for corporations to secure new opportunities to exploit sources of profits.

The “imaginary social order” (Pearce, 1976) found in the print media is a product of overreliance on industry insiders as official sources providing the language with which to render corruption intelligible. This reproduction of the claims of primary definers entailed avoiding flak and also othering and, with this, complicity with the claims of powerful international interests. Canadian newspapers reproduce a narrow legalistic definition of corruption and ignore the expansion of corporate power and do not hold corporations to account for their wrongdoing. Moreover, Canadian print media are reproducers of a politics being played out elsewhere, where the rhetorical register promotes conflicting views on why corruption is a problem, how it is produced, and how to combat it. Predominantly, corruption is construed as bribery, which is said to undermine confidence in markets, government institutions (i.e., undercuts democratic processes), and redirects resources into the pockets of greedy and morally bankrupt individuals.

While there are some news items that question the ability or willingness of governments and corporations to address corruption, or raise issue with the claimed benefits of globalization, the dominant coverage is complicit in the rise of an anti-corruption rhetoric that has done more to justify the expansion of corporate power than contribute to the reduction of the corrosion and distortion of public processes and institutions by private interests (Bratsis 2014; Galanis 2021; Nyberg 2021). Corruption is viewed as a problem of the Global South (i.e., others) and various anti-corruption efforts are linked to the moral duties and responsibilities of Canadian governments and corporations to instill good governance and democratic principles in ‘under-developed’ countries via liberalized trade markets. Anti-corruption is portrayed as the need to allow corporations to fully realize their social responsibilities via self-regulation. In this respect, as we demonstrated, Canadian newspapers played a significant role – as a site of struggle channeling the voices of powerful actors – in advancing corporate ethics and social responsibility as part of a broader agenda to avoid formal rules and laws to address corporate wrongdoing. Canadian print media was and is part of the global expansion of corporate power rather than a means by which we might hold corporations to account for their harms and crimes.

## Notes

1. We recognize newsprint media as powerful corporations that have experienced an incresing concentration of ownership in recent decades (Winseck, 2021). Media companies are therefore far from neutral in their decisions to cover certain topics, including corruption stories that possibly implicate the media itself and/or companies within their ownership structure. Our analysis, however, focused on the print media as a *site* of struggle over the meaning of corruption and how it should be responded to – something we outline further below in our discussion of primary definers. Given this scope and the nature of the data, we did not examine whether any of our news items involved allegations of corruption directed at a corproate parent, shareholder or filial.

2. As part of this process, we each independently read the news items and then met as a team to discuss and compare our findings and situate them methodologically and theoretically. This included weekly hours-long meetings from September 2022 through January 2024 in which we identified the rhetoric employed in the various newspaper items, tracked emerging themes using a spreadsheet, and generated concepts for better understanding anti-corruption(ism). We then condensed our themes to three key moments or phases of ‘talk’ about (anti)corruption.
